# Advanced Paternal Age Is Associated with Impaired Neurocognitive Outcomes during Infancy and Childhood

**DOI:** 10.1371/journal.pmed.1000040

**Published:** 2009-03-10

**Authors:** Sukanta Saha, Adrian G Barnett, Claire Foldi, Thomas H Burne, Darryl W Eyles, Stephen L Buka, John J McGrath

**Affiliations:** 1 Queensland Centre for Mental Health Research, The Park Centre for Mental Health, Richlands, Australia; 2 Institute of Health and Biomedical Innovation and School of Public Health, Queensland University of Technology, Kelvin Grove, Australia; 3 Queensland Brain Institute, The University of Queensland, St. Lucia, Australia; 4 Department of Community Health, Brown University, Providence, Rhode Island, United States of America; 5 Department of Psychiatry, The University of Queensland, St. Lucia, Australia; University of Cambridge, United Kingdom

## Abstract

**Background:**

Advanced paternal age (APA) is associated with an increased risk of neurodevelopmental disorders such as autism and schizophrenia, as well as with dyslexia and reduced intelligence. The aim of this study was to examine the relationship between paternal age and performance on neurocognitive measures during infancy and childhood.

**Methods and Findings:**

A sample of singleton children (*n* = 33,437) was drawn from the US Collaborative Perinatal Project. The outcome measures were assessed at 8 mo, 4 y, and 7 y (Bayley scales, Stanford Binet Intelligence Scale, Graham-Ernhart Block Sort Test, Wechsler Intelligence Scale for Children, Wide Range Achievement Test). The main analyses examined the relationship between neurocognitive measures and paternal or maternal age when adjusted for potential confounding factors. Advanced paternal age showed significant associations with poorer scores on all of the neurocognitive measures apart from the Bayley Motor score. The findings were broadly consistent in direction and effect size at all three ages. In contrast, advanced maternal age was generally associated with better scores on these same measures.

**Conclusions:**

The offspring of older fathers show subtle impairments on tests of neurocognitive ability during infancy and childhood. In light of secular trends related to delayed fatherhood, the clinical implications and the mechanisms underlying these findings warrant closer scrutiny.

## Abstract 2

Using a sample of children from the US Collaborative Perinatal Project, John McGrath and colleagues show that the offspring of older fathers exhibit subtle impairments on tests of neurocognitive ability during infancy and childhood.

## Editors' Summary

**Background.:**

Over the last few decades, changes in society in the developed world have made it increasingly common for couples to wait until their late thirties to have children. In 1993, 25% of live births within marriage in England and Wales were to fathers aged 35–54 years, but by 2003 it was 40%. It is well known that women's fertility declines with age and that older mothers are more likely to have children with disabilities such as Down's syndrome. In contrast, many men can father children throughout their lives, and little attention has been paid to the effects of older fatherhood.

More recent evidence shows that a man's age does affect both fertility and the child's health. “Advanced paternal age” has been linked to miscarriages, birth deformities, cancer, and specific behavioral problems such as autism or schizophrenia.

Rates of autism have increased in recent decades, but the cause is unknown. Studies of twins and families have suggested there may be a complex genetic basis, and it is suspected that damage to sperm, which can accumulate over a man's lifetime, may be responsible. A woman's eggs are formed largely while she is herself in the womb, but sperm-making cells divide throughout a man's lifetime, increasing the chance of mutations in sperm.

**Why Was This Study Done?:**

There is good evidence linking specific disorders with older fathers, but the link between a father's age and a child's more general intelligence is not as clear. A recent study suggested a link between reduced intelligence and both very young and older fathers. The authors wanted to use this large dataset to test the idea that older fathers have children who do worse on tests of intelligence. They also wanted to re-examine others' findings using this same dataset that older mothers have more intelligent children.

**What Did the Researchers Do and Find?:**

The researchers gathered no new data but reanalyzed data on children from the US Collaborative Perinatal Project (CPP), which had used a variety of tests given to children at ages 8 months, 4 years, and 7 years, to measure cognitive ability—the ability to think and reason, including concentration, memory, learning, understanding, speaking, and reading. Some tests included assessments of “motor skills”—physical co-ordination.

The CPP dataset holds information on children of 55,908 expectant mothers who attended 12 university-affiliated hospital clinics in the United States from 1959 to 1965. The researchers excluded premature babies and multiple births and chose one pregnancy at random for each eligible woman, to keep their analysis simpler. This approach reduced the number of children in their analysis to 33,437.

The researchers analyzed the data using two models. In one, they took into account physical factors such as the parents' ages. In the other, they also took into account social factors such as the parents' level of education and income, which are linked to intelligence. In addition, the authors grouped the children by their mother's age and, within each group, looked for a link between the lowest-scoring children and the age of their father.

The researchers found that children with older fathers had lower scores on all of the measures except one measure of motor skills. In contrast, children with older mothers had higher scores. They found that the older the father, the more likely was this result found.

**What Do These Findings Mean?:**

This study is the first to show that children of older fathers perform less well in a range of tests when young, but cannot say whether those children catch up with their peers after the age of 7 years. Results may also be biased because information was more likely to be missing for children whose father's age was not recorded.

Previous researchers had proposed that children of older mothers may perform better in tests because they experience a more nurturing home environment. If this is the case, children of older fathers do not experience the same benefit.

However, further work needs to be done to confirm these findings. Especially in newer datasets, current trends to delay parenthood mean these findings have implications for individuals, couples, and policymakers. Individuals and couples need to be aware that the ages of both partners can affect their ability to have healthy children, though the risks for individual children are small. Policymakers should consider promoting awareness of the risks of delaying parenthood or introducing policies to encourage childbearing at an optimal age.

**Additional Information.:**

Please access these Web sites via the online version of this summary at http://dx.doi.org/10.1371/journal.pmed.1000040.

- Mothers 35+ is a UK Web site with resources and information for older mothers, mothers-to-be, and would-be mothers, including information on the health implications of fathering a child late in life
- The American Society for Reproductive Medicine published a Patient Information Booklet on Age and Fertility in 2003, which is available online; it contains a small section called “Fertility in the Aging Male,” but otherwise focuses on women
- The online encyclopedia Wikipedia has a short article on the “Paternal age effect” (note that Wikipedia is a free online encyclopedia that anyone can edit; available in several languages)
- In 2005, the UK Office of National Statistics published a booklet entitled “Perpetual postponers? Women's, men's and couple's fertility intentions and subsequent fertility behaviour” looking at data from the British Household Panel Survey

## Introduction

In recent decades there has been increased attention to health outcomes in the offspring of older fathers. Evidence shows that advanced paternal age (APA) is associated with an increased risk of a wide range of disorders [1]. While not discounting the influence of various age-related psychosocial factors that may translate to differential health outcomes for the offspring of older fathers (e.g., higher socioeconomic status, better education), advances in genomics have refocused attention on the vulnerability of sperm from older fathers to carrying de novo mutations. The development of the germ cell differs between human males and females—there are many more germline cell divisions in the life history of a sperm relative to that of an oocyte [2]. In the female there are 22 mitotic cell divisions that occur in utero. In contrast, after puberty, progenitor sperm stem cells undergo mitotic cell division once every 16 d. By age 20 the progenitor sperm cells have undergone approximately 150 cell divisions. By age 50 this number is 840. Thus, the chance of copy error mutations increases with age in males more dramatically than for females.

Advanced paternal age is associated with increased fetal deaths [3,4] and certain rare congenital syndromes (e.g., achondroplasia) [1,5]. In recent years evidence has accumulated linking APA with a wide range of neurological and neuropsychiatric conditions including Alzheimer's disease [6,7], bipolar disorder [8], dyslexia [9], neural tube defects [10], and epilepsy [11]. A sizeable body of evidence has accumulated linking APA with an increased risk of schizophrenia [12–18]. A recent meta-analysis based on eight studies found that paternal age above 35 was associated with an increased risk of schizophrenia [19]. There is also evidence linking APA to autism spectrum disorders [20–24].

The associations between APA and outcomes such as autism and schizophrenia are of particular interest, as these disorders have recently been associated with genomic structural variation [25–30]. It is feasible that APA-related mechanisms may contribute to genomic structural variation (e.g., copy number variants, microdeletions) [2]. Thus, within the fields of schizophrenia and autism research, there has been an unexpected convergence between epidemiology and molecular biology.

While there is good evidence linking paternal age with several clinically distinct neurodevelopmental disorders, the evidence linking paternal age and other neurocognitive outcomes such as general intelligence is less robust. Earlier studies noted an association between APA and poorer performance on neurocognitive tests [31–34]. This issue has been addressed specifically in a recent study based on male and female Israeli conscripts (age 16–17 y, *n* = 44,175) [35]. The study found independent effects of paternal age on offspring intelligence with the lowest scores associated with both younger and older fathers (inverted “U”-shaped association). This finding is in contrast to the association between maternal age and offspring intelligence, where most studies have reported a linear association between older maternal age and superior neurocognitive ability [36–39].

The aim of the present study was to explore the association between paternal age and a range of neurocognitive measures using a large, prospective birth cohort: the US-based Collaborative Perinatal Project (CPP). Based on the literature linking increased paternal age with a range of developmental anomalies and neuropsychiatric disorders, we hypothesized that the children of older fathers would have lower scores on various tests used to measure neurocognitive ability when assessed at 8 mo, 4 y, and 7 y. While a study based on this same cohort had previously identified that the offspring of older mothers had superior performance on neurocognitive functioning [36], we also took the opportunity to re-examine this hypothesis in the current analyses.

## Methods

### Sample Selection

The Collaborative Perinatal Project (CPP) recruited pregnant women from 12 university-affiliated hospital clinics in the United States of America from 1959 to 1965. The selection method varied from centre to centre, with between 14% and 100% of the registered pregnant women being invited to participate. At centres with less than 100% sampling, women were selected according to various quasi-random rules (e.g., every *n*th woman). Of 132,560 eligible pregnancies, 55,908 pregnancies were included, which was a proportion representative of the original sampling frame [40,41].

In order to reduce the impact of prematurity on the neurocognitive outcome measures, we restricted the sample to offspring born after 37 wk gestation. In order to minimize statistical complexities arising from dependent data, we restricted the sample to (a) singleton pregnancies, and (b) one randomly chosen pregnancy for each woman enrolled in the study.

### Measures of Neurocognitive Function

Study offspring were assessed at regular intervals until age 7 y. Detailed descriptions of the methods used for cognitive assessments have been published elsewhere [36,42]. At 8 mo of age the Bayley Scales for Infant Development were administered [43,44]. Two scores were available: (a) Mental Scale, which assesses aspects of development including sensory discrimination and eye-hand coordination, and (b) Motor Scale, which assessed various aspects of fine and gross motor coordination. At age 4 y the children were administered (a) the Stanford Binet Intelligence Scale, Form L-M (a measure of general intelligence in young children) [45,46], and (b) the Graham-Ernhart Block Sort Test, which assesses conceptual and perceptual motor ability. This test involves increasingly difficult tasks that range from matching simple like-shaped blocks, to sorting blocks according to one or two dimensions (e.g., colour, shape, size) [47]. At age 7 y the children were administered the widely used Wechsler Intelligence Scale for Children (WISC) [48]. Scores for Full Scale, Verbal, and Performance were available for this study. However, the two WISC subscales (Verbal and Performance) were strongly correlated with WISC Full Scale IQ (Pearson correlation = 0.90 and 0.89, respectively), thus only the WISC Full Scale IQ results are presented. The Wide Range Achievement Test (WRAT) scale was also used at the age 7 y follow-up in order to evaluate academic achievements (e.g., the ability to read words, comprehend sentences, spell, and compute solutions to math problems) [49]. Scores for WRAT Arithmetic, Reading, and Spelling were available in this study. Because the WRAT Reading, Spelling, and Arithmetic scores were all strongly correlated (Pearson correlations of 0.65 to 0.89), only the WRAT Reading is presented.

### Statistical Methods

For the primary analyses, we modelled nonlinear associations between parental age and neurocognitive outcomes using a generalized additive model [50]. We used the generalized cross-validation algorithm to select the degree of nonlinearity. To verify the assumptions of the models, we examined the residuals to check (a) their normality and (b) their homoscedasticity (constant variance) against paternal age.

Each parent's age (at the birth of the child) was adjusted for the other parent's age. For the primary analyses, we examined a simple model (Model 1) adjusted for offspring sex, other parent's age, mother's race, weeks of gestation, and child's age at testing (which varied slightly at the 8 mo, 4 y, and 7 y follow-ups). In order to explore if various socioeconomic variables influenced the strength of the association, a second model (Model 2) also included additional adjustments related to maternal marital status, family socioeconomic status and parental mental health. Socioeconomic status was measured by a composite index that averaged centiles derived from maternal and paternal education and occupation, as well as family income [51].

Because maternal and paternal age were strongly correlated (Pearson correlation = 0.80), we checked the models for colinearity using the variance inflation factor [52]. The variance inflation factors were roughly three for paternal and maternal age in all models. This value is well below the suggested threshold of ten [52], and hence we modelled both ages together.

The results of the primary analyses are displayed graphically, with the nonlinear model fitted for both maternal and paternal age (and 95% confidence intervals [CIs]). The variance explained (adjusted *R*-squared) and the *p*-values for each of the primary analyses are also shown in tabular form. Nonlinear models do not lend themselves to simple quantitative descriptions (e.g., statements such as “the outcome variable falls by a certain number of units for every additional 5 years of paternal age” cannot be made for nonlinear relationships). In order to facilitate interpretation of the primary analyses, we also provided estimates (and 95% CIs) for each outcome variable at two paternal ages (20 and 50 y).

As secondary analyses, we examined the association between paternal age and offspring neurocognition according to various strata of maternal age. This removes widely diverse effects due to maternal age on neurocognitive outcomes by estimating the effect of paternal age in subgroups where mother's ages were highly comparable. We identified cohort members where maternal ages fell within roughly 5 y age strata: <20, 20–24, 25–29, 30–34, 35–39, 40+. For these secondary analyses, we also chose a more stringent test of the association between the variables of interest. For each of the neurocognitive variables, we stratified the sample by sex, age, and race and then dichotomized the sample into a low-achievers group, defined as the lowest 10% of scores in each sex, age, and race group, versus the remaining 90% of the group. We calculated the adjusted odds ratio for being in the low achievers group for a 5 y increase in paternal age using conditional logistic regression.

All *p-*values were two-sided and statistical significance was set at 0.05. We used the *mgcv* library in *R* to fit the generalized additive models [53] and SAS PROC PHREG for the conditional logistic regression [54].

## Results

There were 55,740 singleton pregnancies. Of these, 12,297 children were excluded because of (a) missing maternal and/or paternal age (1,542), (b) having indeterminate or unspecified sex (1,050), or (c) gestational age that was missing or less than 37 wk (9,705). After randomly selecting one live-born offspring per study mother, this left a total of 33,437 study offspring (17,148 males) available for the main analyses. Of these, 51% of the mothers were white, 39% black, and the remaining 10% were Asian and other racial groups. Finally, 6,355 children were missing information about age at testing at 8 mo, while 9,930 were missing age at testing at 4 y, and 9,109 were missing age at testing at 7 y. Those with missing paternal age were significantly more likely to have missing outcome variables at 8 mo, 4 y, and 7 y (each *p* < 0.001).

**Figure 1:**
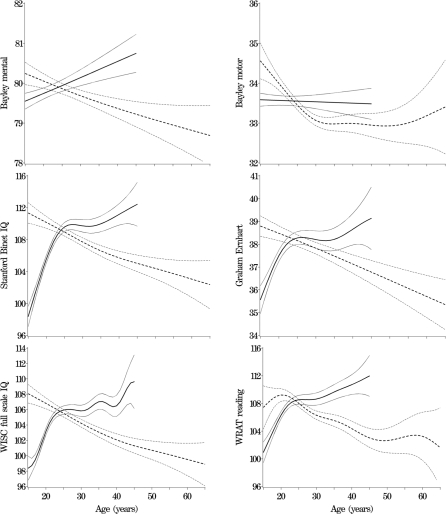
Primary Analyses: Model 1—Adjusted for Other Parent's Age, Mother's Race, Gestational Age, and Child Gender Solid lines ranging from 15 to 45 y for maternal age, dotted lines ranging from 15 to 65 y for paternal age. Nonlinear model fit with 95% CIs.

**Figure 2:**
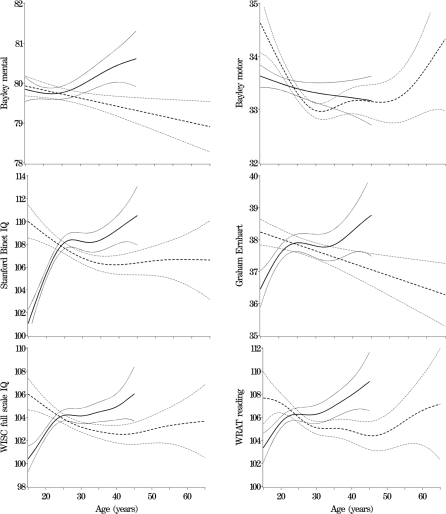
Primary Analyses: Model 2—Adjusted for Other Parent's age, Mother's Race, Gestational Age, and Child Gender, Socioeconomic Index, Marital Status, and Maternal and Paternal Mental Illness Solid lines ranging from 15 to 45 y for maternal age, dotted lines ranging from 15 to 65 y for paternal age. Nonlinear model fit with 95% CIs.

Table 1 shows descriptive statistics for paternal and maternal age and differences in parental age. On average, fathers were 3 to 4 y older than mothers, but the differences in parental age varied widely. Concerning the primary analyses, there was a statistically significant association between advanced paternal age and *inferior* performance on all neurocognitive tests (all *p* < 0.001) except for Bayley Motor score (Model 2, *p* = 0.104) (see Table 2). Concerning the influence of maternal age, there were statistically significant associations between advanced maternal age and *superior* performance on all measures. Figures 1 and 2 show the mean adjusted score for paternal and maternal age for the outcome variables based on Models 1 and 2 respectively. Apart from the direction of the association between maternal and paternal age, the association between maternal age and the outcome variables at ages 4 and 7 y was curvilinear (generally steep at younger ages, then less steep at older ages), in contrast to the near-linear association with paternal age. Post-hoc analyses examining the goodness-of-fit of nonlinear versus linear models indicated that two of the variables were adequately capture by simple linear models (Bayley Mental score and Graham Ernhart Block Sort Test), but that nonlinear models were best suited for all other variables (unpublished data). Table 3 shows the estimated scores (and 95% CIs) for two paternal ages (20 and 50 y) based on the nonlinear modelling used in the primary analyses. For Model 2, the adjusted R-squared ranged from 2.4% (Bayley Motor) to 29.5% (WISC Full Scale IQ).

**Table 1:**
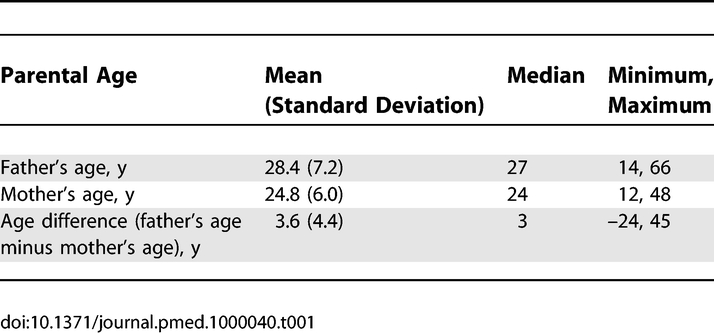
Descriptive Statistics of Maternal and Paternal Age, and Parental Age Difference (*n* = 33,437)

**Table 2:**
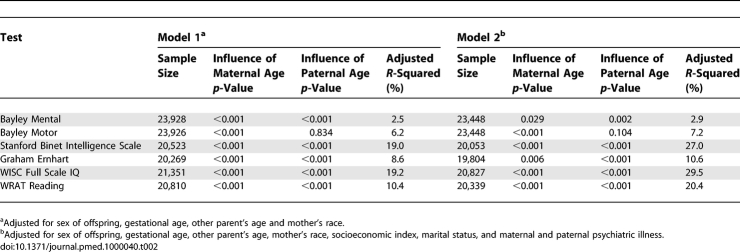
Primary Analyses: Summary Table for the Nonlinear Model Fits for Models 1 and 2

**Table 3:**
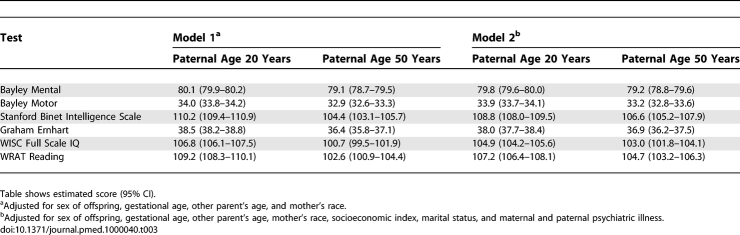
Primary Analyses: Estimates for Two Paternal Ages Based on the Nonlinear Model Fits for Models 1 and 2

Concerning the secondary analyses, the odds ratio (Model 2) for being in the lowest decile for each neurocognitive variable was significantly associated with elevated paternal age for three of the neurocognitive measures (Bayley Motor, Graham Ernhart Block Sort Test, WISC Full Scale IQ), with trend level association identified for the other three measures (Bayley Mental, Stanford Binet Intelligence Scale, WRAT Reading) (Table 4).

**Table 4:**
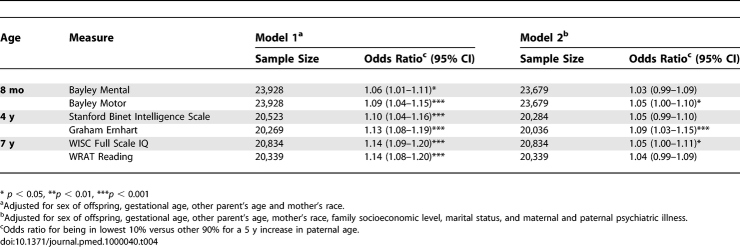
Secondary Analyses: Associations between Increasing Paternal Age and Neurocognitive Measures in Children, Results from Logistic Regression Analyses Using Subgroups of Women with Similar Ages

## Discussion

We report, to our knowledge for the first time, that the offspring of older fathers show impairments on a range of neurocognitive tasks during infancy and childhood. The pattern of findings was relatively consistent across ages and across neurocognitive domains, with near-linear declines found in most of the measures. When the data were examined with a more stringent definition of cognitive impairment (scores in the lowest 10%), a significant relationship between APA and impaired neurocognition was found for three of the six outcome variables, with trend level associations found for the remaining three variables. These findings persisted after adjustment for a range of socioeconomic variables and for parental mental health. In striking contrast to the findings for APA, the association between advanced maternal age and performance on neurocognitive tasks was in the opposite direction.

The findings differ somewhat from those reported by Malaspina et al. [35], who reported on four different measures related to cognitive ability in teenagers (age 16–17y). In that study the offspring of both younger (<20 y) and older fathers (>40 y) had impaired neurocognitive performance compared to those with fathers in the other age strata. However, differences between the Malaspina et al. study and the current study with respect to the psychometric measures and the age of the offspring make direct comparisons difficult. As expected, the current study also identified an association between advanced maternal age and superior performance on the neurocognitive tests, in keeping with some [36–39] but not all studies [35].

The association between APA and reduced neurocognitive ability may have important implications for clinical outcomes previously linked to APA. While not all individuals with autistic spectrum disorders have impaired intelligence, many have specific learning disabilities and/or intellectual handicap [55]. With respect to schizophrenia, systematic reviews and meta-analyses have shown a reliable, medium-sized impairment in premorbid intelligence associated with this disorder [56,57]. For example, Woodberry et al. [57] reported that years before the onset of psychotic symptoms, individuals who later developed schizophrenia had IQ scores that, on average, were approximately one-half of a standard deviation below that of healthy comparison participants. Consistent with these findings, a systematic review of the antecedents of schizophrenia based on prospective birth cohorts [58] provided robust evidence that individuals who later develop schizophrenia show deviation during childhood on a range of cognitive measures related to intelligence, motor development, speech and language, and educational outcomes. In particular, cohort members who later developed schizophrenia, as a group, achieved lower scores on intelligence tests in childhood and adolescence than their peers [59–61].

The findings from this study linking APA and impaired cognition may be best conceptualized within the notion of impaired cognitive reserve [62,63]. Just as superior cognitive capacity appears to provide a buffer against dementia [64,65], subtle APA-related impairments in neurocognitive ability may contribute to an increased risk of a diverse range of adverse neurological and neuropsychiatric health outcomes.

The study has several caveats. Nonrandom sample attrition and missing data may influence the generalisability of the findings [41]. Those with missing data on paternal age were more likely to be lost to follow-up. It will be important to examine the variables of interest in cohorts with optimal participant retention and minimal missing data. More importantly, the cohort members were born in the United States during the 1960s, thus the generalisability of the findings with respect to more contemporary cohorts needs to be examined. While it is feasible that various economic and psychosocial factors that can influence childhood developmental trajectories may have changed in recent decades, there is no reason to suspect that the putative biological processes linking APA and adverse health outcome would have varied over this time frame. Finally, it is important to note that these analyses investigated neurocognitive outcomes only until age 7 y, and it is feasible that the offspring of older fathers “catch up” during later childhood. How the subtle neurocognitive features associated with APA translate into later educational and mental health outcomes across the lifespan remains to be determined.

With respect to the mechanism of action underpinning these findings, several hypotheses warrant further scrutiny. While twin studies have demonstrated that cognitive ability and brain structure are heritable [66,67], studies based on sibships within the CPP have also confirmed that socioeconomic factors play a role in mediating the heritable aspects of intelligence [68]. With respect to paternal age, a broad range of socioeconomic factors improve with increasing age, thus most commentators believe that the offspring of older parents would have better access to health and educational services compared to the offspring of younger parents (who tend to have lower education and poorer income) [69]. For example, Fergusson and Lynsky [38] found that offspring of younger mothers tended to be born into relatively poorly educated and socially disadvantaged families. These authors commented that children born to young mothers were exposed to less nurturing and more changeable home environments. One would expect that such mechanisms would also operate with respect to paternal age. Clearly, our findings linking APA with impaired neurocognitive development cannot be readily explained by these social mechanisms.

Mechanisms related to the development of the male germline warrant consideration [70]. Each time the cell divides, the replication of the genome introduces the possibility of copy error mutations. In humans it has been confirmed that sperm from older men have significantly more mutations [2,71,72]. Levels of DNA proofreading and repair enzymes also decline as a function of APA [16] and DNA fragmentation increases [73], further compromising the integrity of gene replication. Apart from genetic changes (i.e., changes in DNA basepair sequence), APA may also involve abnormal epigenetic mechanisms [74–76].

Unravelling the molecular mechanisms underlying the association between APA and adverse health outcomes will be a substantial task for the biomedical research community. The precise location and nature of these mechanisms will probably vary substantially from offspring to offspring. It is unlikely that they will “map” neatly to a few loci, nor probably to one mechanism (e.g. genetic, epigenetic). With respect to genetic mechanisms, these may include single nucleotide mutations, or various types of genomic rearrangements (e.g., microdeletions, tandem and trinucleotide repeat expansions, microduplication or higher order expansions, aneuploidy). Animal experiments based on inbred rodent strains may provide the most efficient way to explore genetic and epigenetic factors mediating APA and brain development. Comparable to “forward genetics” platforms based on chemical mutagens [77,78], rodent-based APA models could provide an age-related mutagenesis experiment that has epidemiological face validity [79].

The observation linking APA with risk of schizophrenia has led to the hypothesis that APA-related mechanisms are contributing de novo mutations, which could explain the persistence of schizophrenia in the population in spite of reduced fertility and/or fecundity associated with this disorder [80]. APA-related mechanisms could accumulate over several generations, with the full clinical phenotype “breaking through” only after a critical threshold of certain mutations have accumulated [81,82]. In light of secular trends related to delayed parenthood [83], and in light of the potential for APA-related mechanisms to accumulate over several generations, the association between APA and subtle deficits in neurocognitive outcomes warrants closer scrutiny. While most of the neurocognitive differences were small at the individual level, these could have important implications from a public health perspective [84].

## Abbreviations

APA: advanced paternal age
CI: confidence interval
CPP: Collaborative Perinatal Project
WISC: Wechsler Intelligence Scale for Children
WRAT: Wide Range Achievement Test
